# ROBOTIC VERSUS LAPAROSCOPIC ROUX-EN-Y-GASTRIC BYPASS: A RETROSPECTIVE STUDY IN A SINGLE CENTER

**DOI:** 10.1590/0102-672020230038e1756

**Published:** 2023-09-15

**Authors:** Fernando de BARROS, Ana Beatriz Monteiro FONSECA, Amanda Sebestjen Balogh KISS, Camilla Ferreira BRAGA, Filipe Roza DA-SILVA, Yumi Honda REGONATI

**Affiliations:** 1 Universidade Federal Fluminense, Department of General and Specialized Surgery – Niterói (RJ), Brazil; 2 DASA, Robotic Surgery – Rio de Janeiro (RJ), Brazil; 3 Universidade Federal Fluminense, Statistics Department – Niterói (RJ), Brazil

**Keywords:** Bariatric surgery, Gastric bypass, Robotic surgical procedures, Cirurgia bariátrica, Derivação gástrica, Procedimentos cirúrgicos robóticos

## Abstract

**BACKGROUND:**

Bariatric surgery is the best treatment option for patients with obesity. As a result of the advancement of technology, the robotic gastric bypass presents promising results, despite its still high costs.

**AIMS:**

The aim of this study was to compare patients submitted to a robotic *versus* a laparoscopic gastric bypass at a single center by a single surgeon.

**METHODS:**

This retrospective study collected data from the medical records of 221 patients (121 laparoscopic procedures versus 100 with daVinci platform). The variables analyzed were sex, age, body mass index, comorbidities, surgical time, length of stay, and complications.

**RESULTS:**

The mean surgical time for patients in the robotic group was shorter (102.41±39.44 min *versus* 113.86±39.03 min, p=0.018). The length of hospital stay in robotic patients was shorter (34.12±20.59 h versus 34.93±11.74 h, p=0.007). There were no serious complications.

**CONCLUSIONS:**

The group submitted to the robotic method had a shorter surgical time and a shorter hospital stay. No difference was found regarding strictures, bleeding, or leakage.

## RESUMO

**RACIONAL:**

A cirurgia bariátrica é a melhor opção de tratamento para pacientes portadores de obesidade. Em decorrência do avanço da tecnologia, o bypass gástrico robótico apresenta resultados promissores, apesar de seus custos ainda elevados.

**OBJETIVOS:**

Comparar pacientes submetidos a *bypass* gástrico robótico versus laparoscópico em um único centro por um único cirurgião.

**MÉTODOS:**

Estudo retrospectivo com coleta de dados dos prontuários de 221 pacientes (121 procedimentos laparoscópicos *vs* 100 com plataforma daVinci). As variáveis analisadas foram sexo, idade, IMC, comorbidades, tempo cirúrgico, tempo de internação e complicações.

**RESULTADOS:**

O tempo cirúrgico médio dos pacientes do grupo robótico foi menor (102,41 ± 39,44 min. *vs* 113,86±39,03 min, p=0,018). O tempo de internação em pacientes robóticos foi menor (34,12±20,59 h *vs* 34,93±11,74 h, p=0,007). Não houve complicações graves.

**CONCLUSÕES:**

O grupo submetido ao método robótico apresentou menor tempo cirúrgico e menor tempo de internação. Nenhuma diferença foi encontrada na amostra em relação a estenoses, sangramento ou vazamento.

## INTRODUCTION

Bariatric surgery grows along with the exponential increase in obesity worldwide, and it is the best therapeutic option for this disease^7,11^. New treatments and new technologies are emerging in medical care to prevent and manage obesity. Especially in patients with severe obesity or patients at a higher risk, advanced treatments and new technologies may help this population to achieve good results and increase security in the management. Revisional surgery is another situation that could have benefits from new technologies^18^.

In the mid-1990s, laparoscopic Roux-en-Y gastric bypass (LRYGB) emerged and soon became widespread with excellent results^21^. Recently, robotic Roux-en-Y gastric bypass (RRYGB) has emerged as a breakthrough^8,10,13,14^. However, robotic costs are still significantly higher compared to the laparoscopic procedure. Mainly for this reason, some surgeons are still skeptical about the cost-benefit of the robotic platform in bariatric surgery. Besides that, bariatric surgery has already good results with a low ratio of complications^12^. So, will the robot bring better results to our patients in primary bariatric surgery?

The studies available are still quite heterogeneous, showing not only some advantages of robotic surgery such as a shorter hospital stay but also disadvantages such as longer surgical time and higher cost and complications^1,10,15^. 

This study aimed to retrospectively analyze patients submitted to LRYGB *versus* RRYGB, performed in a single center, by a single surgeon over a 2-year period.

## METHODS

All search procedures in this work were conducted in accordance with the institutional ethical guidelines for human studies following all the principles for medical research involving human subjects. The study was approved by the Ethics Committee of the São Lucas Hospital board (n° 67889617.3.0000.5533). Informed consent was obtained from all patients.

This is a retrospective study, through the analysis of medical records with data collection of patients, who underwent RRYGB (daVinci Si® Platform) and LRYGB in a single center and performed by a single surgeon. 

All patients were included in the following criteria for an indication for bariatric surgery: grade II obesity with comorbidities or grade III obesity. The following data were collected: sex, age, body mass index (BMI), comorbidities, time of surgery, length of hospital stay, drain debt, and postoperative complications (i.e., bleeding, fistula, thrombosis, and readmission).

The anastomoses were stapled using the Signia stapler (Medtronic®), in both groups: gastric pouch between 50 and 70 mL, using purple loads, and both anastomoses with beige loads (Medtronic®). Reinforcement sutures were not performed, and biological material was not used in the loads. The dissection system used in the RRYGB group was the ultrasonic robotic arm (Intuitive®), and in the LRYGB group, we used the Sonicision® (Medtronic®). All mesenteric defects were closed with 3.0 polypropylene. We put a drain routinely in all patients.

All patients had the same postoperative routine: liquid diet and walking until 8 h after the procedure. Restricted liquid diet for 15 days followed by a pasty diet for another 15 days. Enoxaparin® at a dose of 60 mg was used immediately after the procedure and for 10 days after hospital discharge. The criteria to go home were the same in both groups: acceptance of a liquid diet, wandering by themselves, and pain controlled.

The chi-square test was used to verify the association between two qualitative variables. The scatter diagram and Pearson’s linear correlation coefficient were used to verify the existence of an association between the quantitative variables. In this case, a hypothesis test was also applied to identify whether the observed correlation value was significantly different from zero. The Shapiro-Wilk test was used to assess the adequacy of the assumption of normality, for the distributions of quantitative variables. The Mann-Whitney test was adopted for the comparison between the groups regarding quantitative variables, in the absence of adequacy to the normal model. The significance level adopted in the analyses was 5%.

## RESULTS

A total of 221 patients were analyzed: 121 RRYGB (54.3%) and 100 LRYBG (45.7%). Patients submitted to RRYGB had a mean age of 40.57±10.64 years, mean weight of112.35±20.99 kg, mean height of 164.14±9.03 cm, and mean BMI of 42.74±5.91 kg/cm². Patients submitted to LRYGB had a mean age of 39.16±8.60 years (p=0.15), mean weight of 115.33±20.71 kg (p=0.19), mean height of 166.13±8.72 cm (p=0.26), and mean BMI of 41.64 ± 5.04 kg/cm² (p=0.37).

**Figure 1:**
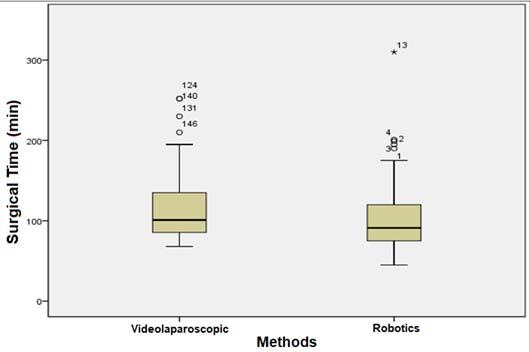
Comparison of surgical time between laparoscopic Roux-en-Y gastric bypass B and robotic Roux-en-Y gastric bypass groups (p=0.018).

**Figure 2:**
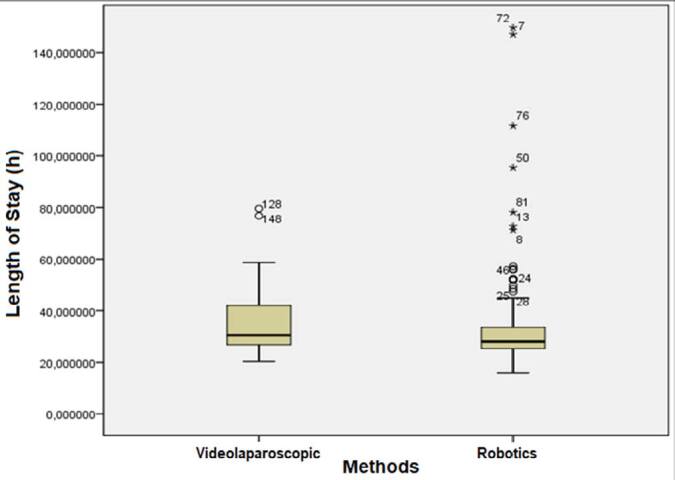
Comparison of length of stay between laparoscopic Roux-en-Y gastric bypass and robotic Roux-en-Y gastric bypass groups (p=0.007).

The mean surgery time of patients submitted to RRYGB was shorter, i.e., 102.41±39.44 min *versus* 113.22±39.03 min for patients submitted to LRYGB (p=0.018) (Figure 1). The drain debt (mL) had no statistically significant difference between the groups: 56.21±65.24 mL in patients undergoing RRYGB *versus* 62.72±62.06 mL in the LRYGB group (p=0.225). The length of hospital stays of patients submitted to RRYGB was shorter as well 34.12±20.59 h *versus* 34.93±11.74 h in the LRYGB group (p=0.007) (Figure 2). There were no serious complications such as bleeding, fistula, stenosis, or thrombosis in any of the patients analyzed.

**Figure 3:**
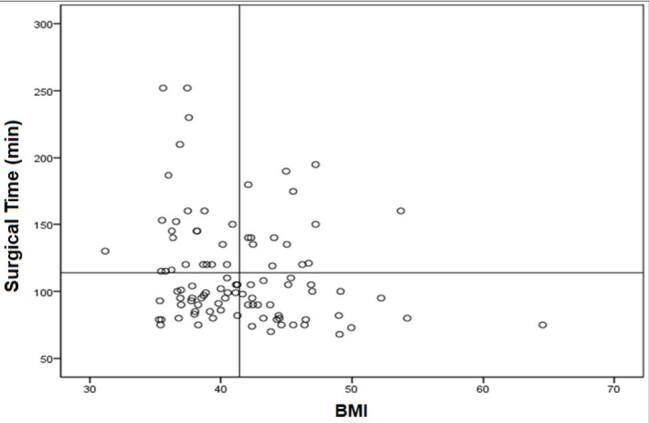
Linear correlation between surgical time and body mass index in the laparoscopic Roux-en-Y gastric bypass group (r=-0.20 and p=0.04).

**Figure 4:**
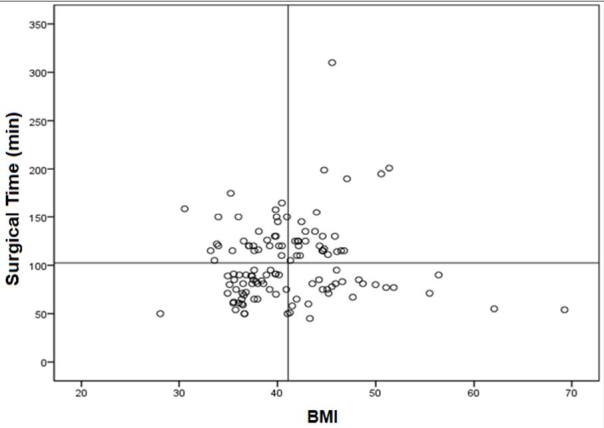
Linear correlation between body mass index and surgery time and body mass index in the robotic Roux-en-Y gastric bypass group (r=0.6 and p=0.55).

There was no linear correlation between the BMI and the surgical time of the patients (r=-0.44 and p=0.51), but in the LRYGB group, the higher the BMI had a longer surgical time (r=-0.20 and p=0.04), unlike in the RRYGB group (r=0.6 and p=0.55) (Figures 3 and 4).

BMI: body mass index.

BMI: body mass index.

Regarding the length of hospital stay, there was no linear correlation between the patients analyzed (r=-0.009 and p=0.89) or in separated analyzed LRYGB (r=-0.107 and p=0.29) and RRYGB (r=0.028 and p=0.76).

## DISCUSSION

### Surgical time

LRYGB has been established for decades. Currently, the robotic procedure has been growing worldwide^6^. Some studies show that robotic surgery does not increase the surgical time or complication rate after surgical learning curve^1^. In general, this can be justified mainly for more complex cases, such as super obese patients^14^. However, some studies carried out in large databases have shown that robotic surgery has a longer operative time^4^. It is noteworthy that despite the large samples in these kinds of studies, there is a high heterogeneity because the evaluation comes from teaching hospitals to a high-volume center considered the center of excellence. In this sense, we understand that studies carried out in a single center with a single surgeon, despite a smaller sample, are of great value. In our study, we observed a shorter surgical time with the robotic platform (102.41±39.44 *versus* 113.86±39.03 min, p=0.018).

Another interesting result that needs comment is the surgical time in correlation with BMI. We observed a linear correlation in the LRYGB: the higher the BMI, the longer the surgical time (r=-0.20 and p=0.04). However, the BMI did not make any difference in the RRYGB group (r=0.6 and p=0.55). One of the reasons may be the benefits of robotic in patients with a higher BMI. The platform allows the surgeon to lift the abdominal wall and to make the sutures more comfortable, precise, and faster in small spaces^18^.

### Learning curve

The learning curve for RRYGB has been lower compared to LRYGB, especially because surgeons, when starting robotic surgery, already have a great experience with minimally invasive gastric bypass^2^. Vilallonga etal. reported 20 cases on the robotic platform to be considered learning curve^20^. Bustos etal. stated that performing the first cases with manual anastomosis can shorten the learning curve^3^. Evidently, as the learning curve progresses, the surgical time is supposed to be shorter^3^. All robotic surgeries performed in this study were performed after 50 robotic procedures by the surgeon.

### Length of hospital stay

The length of hospital stay is one of the most analyzed variables in studies comparing laparoscopic versus robot in gastric bypass. Most studies found a shorter hospital stay in patients operated on the robotic platform. In a meta-analysis, Markar etal. reviewed 41 articles and found a shorter hospital stay in the robot-operated group^9^. Economopoulos etal. analyzed 162 articles with 5145 patients and found a trend toward shorter hospital stays in robotics groups^5^. In a similar study to our own, Stefanidis etal., despite having a much longer surgical time with the robotic platform, also observed a shorter hospital stay in this group^19^. Our sample had a shorter hospital stay in the RRYGB group (p=0.07).

### Drain debt

We routinely use a drain to make a diagnosis of early postoperative complications. Senellart etal. in their study identified a small increase in their bleeding rate in laparoscopic surgery compared to robotic surgery, which was justified in the study because of a better vision when performing manual anastomoses with robotic surgery^17^. In our study, there was no statistically significant difference between the drain debt in the LRYGB and RRYGB groups. The volume in the drain had no difference even when we compared age, BMI, and comorbidities. However, we need to consider that the study was not randomized.

### Complications (i.e., bleeding, stenosis, fistula, and death)

Sebastian etal. identified a lower leakage rate with the robotic technique: 0.5% *versus* 0.9% compared with the laparoscopic approach. The data are consistent with those in other studies found in the literature^16^. Markar etal., in their study with 1686 patients, found a significantly reduced incidence of anastomotic stenosis in the robotic group (POR=0.43; 95%CI=0.19–0.98; p=0.04). However, there was no significant difference in postoperative complications^9^. Economopoulos etal., in their study with 5145 patients submitted to gastric bypass techniques using LRYGB and RRYGB, reached the same result as Markar etal.^9^, demonstrating that the robotic approach is a safe alternative for this procedure^5^. In our study, there were no serious complications such as bleeding, fistula, stenosis, thrombosis, or death in any of the patients analyzed in both groups.

Besides some limitations in the study as a small sample and groups not randomized, these primary results showed that RRYGB may have a place in bariatric surgery in some centers. However, the costs are still high and probably will get better with new platforms coming to the market. We did not compare costs because of bias in our systems (MVSOUL®) that charge different items in the accounts; however, there is no doubt that RRYGB was more expensive than LRYGB.

## CONCLUSION

The group submitted to RRYGB had a shorter surgical time and length of hospital stay compared to LRYGB. We did not observe any difference between the groups in terms of readmissions, strictures, bleeding, or leakage.
